# Epidemiology, antimicrobial resistance, and mortality risk factors of carbapenem resistant gram-negative bacteria in hematopoietic stem cell transplantation recipients

**DOI:** 10.3389/fcimb.2022.1098856

**Published:** 2023-01-13

**Authors:** Yan Jia, Yun Li, Yi Liu, Ziyue Yang, Xuefeng Chen, Yanfeng Liu

**Affiliations:** ^1^ Department of Hematology, Xiangya Hospital, Central South University, Changsha, China; ^2^ Department of Gastroenterology, Xiangya Hospital, Central South University, Changsha, China; ^3^ Department of Oncology, National Health Commission (NHC) Key Laboratory of Cancer Proteomics, Laboratory of Structural Biology, Xiangya Hospital, Central South University, Changsha, China; ^4^ National Clinical Research Center for Geriatric Disorders, Xiangya Hospital, Central South University, Changsha, China; ^5^ Department of Hematology, The Third Xiangya Hospital of Central South University, Changsha, China

**Keywords:** carbapenem resistant gram-negative bacteria, hematopoietic stem cell transplantation, drug resistance, mortality, risk factors

## Abstract

**Introduction:**

Carbapenem resistant gram-negative bacteria (CRGNB) infection is more and more frequent in patients after hematopoietic stem cell transplantation (HSCT), and the prognosis is very poor. The purpose of this study was to investigate the clinical characteristics and risk factors for mortality with CRGNB infection in HSCT recipients, and to provide useful information for guiding the application of antibiotics and improving the prognosis in the future.

**Methods:**

Electronic medical records of CRGNB infected patients who underwent HSCT in Xiangya Hospital from January 1, 2015 to June 30, 2022 were collected. At the same time, 1:1 case-control matching was performed according to gender, age and disease type. The epidemiological characteristics and drug resistance of patients with CRGNB infection and non-CRGNB infection were compared. Logistic regression and Cox regression analysis were used to determine the risk factors for CRGNB acquisition and death respectively, and a prediction model of overall survival was constructed by R language.

**Results and Discussion:**

The crude infection rate of CRGNB in HSCT recipients was 7.42%, and the mortality rate was 47.1%. CRGNB was resistant to most commonly used antibiotics. Time interval from diagnosis to transplantation >180 days (HR=7.886, 95% CI 2.624-23.703, *P*=0.000), septic shock (HR=6.182, 95% CI 2.605-14.671, *P*=0.000), platelet count < 20 × 10^9^/L (HR=2.615, 95% CI 1.152-5.934, *P*=0.022) and total bilirubin > 34.2 μmol/L (HR=7.348, 95% CI 2.966-18.202, *P*=0.000) at the initial stage of infection were 4 independent risk factors associated with mortality. CRGNB infection has become a serious threat to HSCT recipients. Clinicians should pay high attention to it and actively seek personalized treatment strategies suitable for local medical conditions.

## Introduction

With the continuous improvement of technology and the expansion of donor options, hematopoietic stem cell transplantation (HSCT) has become an important, even the best treatment for hematological malignancies, some solid tumors, inherited metabolic disorders and immune diseases (Khaddour et al., 2022). However, HSCT recipients are particularly vulnerable to bacterial infection due to mucosal injury, immunosuppression, prolonged hospitalization, invasive procedures and frequent exposure to antibiotics. In recent years, most regions of the world have shown a trend of epidemiological change from gram-positive bacteria to gram-negative bacteria (GNB) (Omrani and Almaghrabi, 2017). It has been reported that approximately 15% to 74% of bacteremia in HSCT recipients is caused by GNB (Mikulska et al., 2014). In addition, several studies have confirmed that GNB infection is strongly associated with poorer prognosis. For example, Poutsiaka DD et al. reported that the mortality of HSCT recipients experiencing GNB bloodstream infection was about 59% (Poutsiaka et al., 2007). Ortega M et al. and Mikulska M et al. also found that the mortality of HSCT recipients on the 7th and 30th day after GNB bacteremia was 17%-22% and 24%-31%, respectively (Ortega et al., 2005; Mikulska et al., 2012). What’s worse, in the past two decades, the resistance rate of GNB to antibiotics has been increasing, and the emergence of multi-drug resistant (MDR), extensively-drug resistant (XDR), and pandrug resistant (PDR) bacteria has also become more and more common (Sahitya et al., 2021). Carbapenems were once considered as the last line of defense against GNB, whereas the rising drug resistance rate poses a huge challenge to researchers. Carbapenem resistant gram-negative bacteria (CRGNB) is listed as a public health threat prioritized by the Centers for Disease Control and Prevention of the United States and World Health Organization (Babiker et al., 2021).

At present, CRGNB infection has become one of the most common complications and causes of death for HSCT recipients, and the economic burden and mental stress brought by it have a negative impact on patients’ families and even the whole society. Although a number of epidemiological and clinical data related to infection in this special population have been published previously, most have focused on carbapenem-resistant Enterobacteria (CRE). However, some studies demonstrated that infections caused by other CRGNBs, such as Pseudomonas aeruginosa and Acinetobacter, are growing at an extremely fast speed, even surpassing CRE as the most dominant CRGNB isolates among transplant patients in some regions (Babiker et al., 2021).

This study analyzed the composition, distribution, drug resistance and prognosis of CRGNB infection in HSCT recipients in the hematological ward of a large general hospital in Central-south China in the past seven years, and paid special attention to the risk factors related to CRGNB acquisition and death. It provides valuable information for comprehensively understanding the characteristics of post-transplant infection, adjusting anti-infection treatment strategies and improving the prognosis of patients.

## Materials and methods

### Study design and population

The electronic medical record information of patients with CRGNB infection who underwent HSCT in Xiangya Hospital of Central South University (Changsha, China) from January 1, 2015 to June 30, 2022 was collected, and a 1:1 retrospective case-control matching according to gender, age, disease type and other features was conducted (if more than one control patient met the inclusion criteria at the same time, the patient closest to the case without missing data was retained). Clinical characteristics included but were not limited to infection sites, antibiotic use, blood routine/blood biochemical/microbiological laboratory data within 24 hours of infection, acute graft-versus-host disease (aGVHD), septic shock, mechanical ventilation, average hospital stay, survival rate, etc. The follow-up time was 100 days after infection.

Patients younger than 14 years and older than 65 years were excluded. The diagnosis of acute myeloid leukemia, acute lymphoblastic leukemia, myelodysplastic syndrome, aplastic anemia, and lymphoma referred to relevant guidelines (Red Blood Cell Disease (Anemia) Group et al., 2017; Chinese Society of Hematology and Chinese Medical Association, 2019; Hoppe et al., 2020; Hematology Oncology Committee et al., 2021; Leukemia & Lymphoma Group et al., 2021).

### Ethics statement

This was a retrospective case-control study with all data from the electronic medical record system. The institutional review board of Xiangya Hospital endorsed this project and approved the waiver of informed consent from patients (approval number: 2019030162). The authors ensure that the patient’s privacy is not disclosed.

### Definitions

CRGNB mainly included carbapenem resistant- Pseudomonas aeruginosa (CRPA), Acinetobacter baumannii (CRAB), Enterobacteriaceae (CRE) and Stenotrophomonas maltophilia (Doi, 2019). The types of CRE infection were classified according to the National Healthcare Safety Network criteria (Shields et al., 2016). Carbapenem resistance was defined as an ertapenem minimum inhibitory concentration (MIC) ≥ 2 μg/mL and meropenem and/or imipenem MIC ≥ 4 μg/mL. Bacterial infection was defined as the occurrence of inflammatory reaction symptoms in the patient and the isolation of pathogenic microorganisms from blood or other normally sterile sites and exclusion of contamination and colonization (Horan et al., 2018). Infections occurred 48 hours after admission were considered hospital-acquired (Liu et al., 2022). Inappropriate empiric antimicrobial therapy was defined as the absence of microbial-sensitive antibiotics within 48 hours of specimen acquisition (Wan et al., 2017). Antibiotic combination therapy was defined as simultaneous use of two or more antibiotics for at least 48 hours, including inhaled products (only in cases of respiratory infection) (Chen et al., 2021). Neutrophil engraftment was defined as neutrophil > 0.5×10^9^/L for at least 3 consecutive days. Platelet engraftment was defined as platelet > 20×10^9^/L for at least 7 consecutive days without blood transfusion (Huo et al., 2020). Acute GVHD was defined as an inflammatory response in the skin, gastrointestinal tract and liver that occurred within 100 days after transplantation (Stem Cell Application Group et al., 2020).

### Antibiotics use and immunosuppressive regimen

Antibiotic prophylaxis: Oral nystatin (50 wu, 3 times a day) and gentamicin (8 wu, 3 times a day) were used for intestinal preparation 5 days before pretreatment chemotherapy. Carbapenems (meropenem/imipenem) or cephalosporins were usually given to prevent bacterial infection if a patient developed agranulocytosis (or agranulocytosis expected within 48 hours) after the start of pretreatment chemotherapy. Antibiotic therapy: When patients were infected, they were usually treated with carbapenem alone or combined with tigecycline depending on their clinical manifestations. In the later stage, with the engraftment of donor stem cells and the relief of infection symptoms, antibiotics were de-escalated to β-lactamase inhibitor compound preparation (such as piperacillin-tazobactam, cefoperazone-sulbactam) or quinolone (Chinese Society of Hematology et al., 2020). All patients received triple immunosuppression (tacrolimus or cyclosporine A, prednisone and mycophenolate mofetil), and some also received ruxolitinib or balliximab.

### Isolate collection and microbiological investigation

Under the premise of strict aseptic procedures, the patient’s blood (venous blood from 2 different sites at the same time) or other samples (such as sputum, urine, bronchoalveolar lavage fluid, pleural/abdominal effusion, etc.) were collected in special culture bottles and immediately sent to microbiology laboratory for bacterial culture. The bacterial strains were identified by VITEK 2 compact automatic bacterial analyzer, the antimicrobial susceptibility was determined by MIC method and Kirby Bauer diffusion method.

### Construction and validation of prediction model for overall survival

First, independent risk factors associated with death were identified by Cox univariate and multivariate analyses, and used as clinical variables. 100-day OS was used as clinical outcome endpoint. Subsequently, a nomogram model was constructed by R software 3.3.0. The consistency index (C-index) was used to compare the prediction result of nomogram with the actual observation data, and a calibration plot was used to visualize the prediction model, so as to evaluate the sensitivity and specificity.

### Statistical analysis

The continuous variables were presented as median and interquartile range (IQR), and comparison between groups was performed by Mann Whitney U-test. The categorical variables were presented as absolute values and percentages, and comparison between groups was performed by Chi square test or Fischer exact test. The nomogram prediction model was constructed using the rms software package in R language. The receiver operating characteristic (ROC) curve was plotted and the area under the curve (AUC) was calculated to evaluate the prediction accuracy of this model. Kaplan-Meier analysis and Log-rank test were used to compare the survival rates of patients with or without risk factors. *P* < 0.05 was considered statistically significant. All statistical analyses were performed through IBM SPSS 24.0.

## Results

### Clinical characteristics

From January 1, 2015 to June 30, 2022, a total of 1173 cases of HSCT were performed in our hospital, of which 87 were included in this study. The crude CRGNB infection rate was 7.4%, including 49 males (56.3%) and 38 females (43.7%), with a mean age of 35 (24.5, 48) years. The main primary diseases were acute lymphoblastic leukemia (28.7%), acute myeloid leukemia (26.4%), aplastic anemia (17.2%), myelodysplastic syndrome (16.1%) and lymphoma (6.9%). The incidence of aGVHD was 51.7%, and 29 recipients (33.3%) received immunosuppressive therapy with ruxolitinib and/or balliximab. Forty infections (46.0%) occurred during agranulocytosis. Fifty-eight patients (66.7%) did not receive appropriate empiric anti-infective treatment.

Compared with matched controls, patients with CRGNB infection had longer engraftment time of neutrophil (*P*=0.012) and platelet (*P*=0.002), lower albumin level in the early stages of infection (*P*=0.005), and worse prognosis (mortality rate 47.1% vs. 25.3%, *P*=0.005). The demographic, laboratory and clinical data were detailed in **Table 1**.

**Table 1:** Baseline characteristics of hematopoietic stem cell transplantation recipients with CRGNB and non-CRGNB.

Characteristic	CRGNB (N=87)	Non-CRGNB (N=87)	*P*
Sex, n (%)			0.644
Female	38 (43.7%)	34 (39.1%)	
Male	49 (56.3%)	53 (60.9%)	
Age, years, median (IQR)	35 (24.5, 48)	34 (23.5, 45.5)	0.406
Primary disease, n (%)			0.155
Acute lymphocytic leukemia	25 (28.7%)	23 (26.4%)	
Acute myelogenous leukemia	23 (26.4%)	34 (39.1%)	
Aplastic anemia	15 (17.2%)	13 (14.9%)	
Myelodysplastic syndrome	14 (16.1%)	7 (8%)	
Lymphoma	6 (6.9%)	2 (2.3%)	
Others	4 (4.6%)	8 (9.2%)	
CMV infection before transplantation, n (%)	1 (1.1%)	0 (0%)	1.000
EBV infection before transplantation, n (%)	18 (20.7%)	8 (9.2%)	0.056
Sibling donor, n (%)	71 (81.6%)	75 (86.2%)	0.536
HLA-identical, n (%)	25 (28.7%)	36 (41.4%)	0.112
Time between diagnosis and transplantation, days, median (IQR)	222 (165, 332)	192 (118, 268)	0.172
Acute GVHD (grade I-II), n (%)	45 (51.7%)	43 (49.4%)	0.879
Special immunosuppressant^#^	29 (33.3%)	18 (20.7%)	0.088
Infection during agranulocytosis	40 (46.0%)	49 (56.3%)	0.225
Inappropriate empiric antimicrobial treatment, n (%)	58 (66.7%)	37 (42.5%)	0.002*
Time of neutrophil engraftment, median (IQR)	14 (12, 16)	13 (11, 15.5)	0.012*
Time of platelet engraftment, median (IQR)	22 (15, 26)	16 (13, 22)	0.002*
Hospital-acquired infection, n (%)	79 (90.8%)	84 (96.6%)	0.213
Indicators within 24 hours of infection			
Neutrophil count, 10^9^/L, median (IQR)	0.95 (0, 3.2)	0.1 (0, 2.25)	0.161
Lymphocyte count, 10^9^/L, median (IQR)	0.1 (0, 0.4)	0.1 (0, 0.5)	0.579
Platelet count, 10^9^/L, median (IQR)	27 (14, 48)	27 (14.5, 61)	0.695
Procalcitonin, μg/L, median (IQR)	0.63 (0.21, 6.63)	0.75 (0.23, 2.41)	0.807
Albumin, g/L, median (IQR)	31.7 (27.9, 34.3)	34.8 (29.3, 37.9)	0.005*
Total bilirubin, μmol/L, median (IQR)	13.9 (9.3, 22.3)	11.8 (7.95, 18)	0.106
Alanine transaminase, median (IQR)	22.3 (13.3, 46.45)	30 (15.95, 58)	0.150
Aspertate aminotransferase, median (IQR)	25 (14.2, 41)	22.6 (15.9, 34.4)	0.625
Creatinine, μmol/L, median (IQR)	61 (53, 76)	62 (53, 76.7)	0.646
Hospital stay, days, median (IQR)	50 (34, 84)	53 (40, 77.5)	0.986
Mortality, n (%)	41 (47.1%)	22 (25.3%)	0.005*

CRGNB, carbapenem-resistant gram-negative bacteria; HLA, human leukocyte antigen; GVHD, graft-versus-host disease; CMV, cytomegalovirus; EBV, epstein-barr virus; IQR, interquartile range.

^#^Special immunosuppressants here mainly refer to ruxolitinib and balliximab.

*P values are statistically significant.

### Overview of CRGNB microbiology

**Figure 1:**
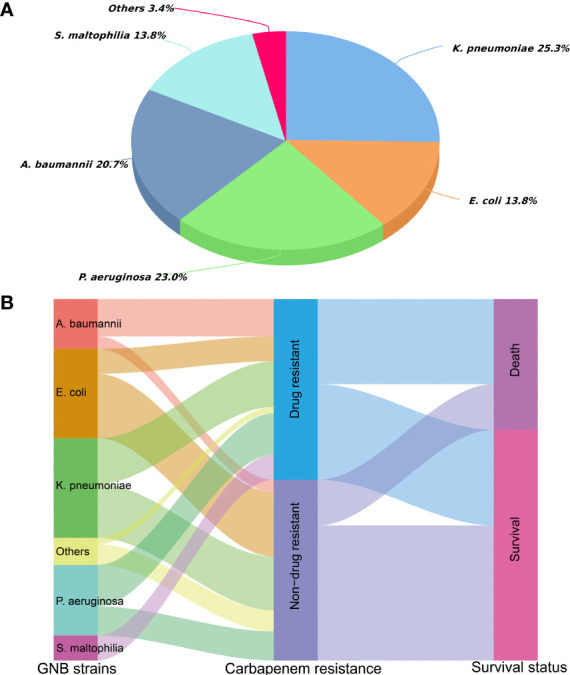
Distribution of carbapenem resistant gram-negative bacteria (CRGNB) and non-CRGNB in hematopoietic stem cell transplantation recipients. **(A)** The classification and percentage of CRGNB. **(B)** The trajectory tracking Sankey diagram among strain type, carbapenem resistance and survival.

Among the 87 isolated CRGNB strains, 56.3% came from lung, 39.1% from the bloodstream, and the rest from urine, skin and soft tissues, pleural/abdominal effusion. **Figure 1A** detailed the classification and percentage of CRGNB. It can be seen that CRKP, CRPA and CRAB were still the main strains of CRGNB infection after HSCT, accounting for more than two thirds of the total. The resistance rate of CRGNB to most commonly used antibiotics (such as quinolones, cephalosporins, sulfonamides and piperacillin-tazobactam) was more than 50%, and it was only relatively sensitive to some aminoglycosides (mainly amikacin here) and tigacycline (TGC). **Figure 1B** showed the trajectory tracking among strain type, carbapenem resistance and survival. The specific comparison between CRGNB and non-CRGNB groups were presented in **Table 2**.

**Table 2:** Overview of CRGNB and non-CRGNB microbiology.

Characteristic	CRGNB (N=87)	Non-CRGNB (N=87)	*P*
Infection sites, n (%)			0.013*
Lung	49 (56.3%)	30 (34.5%)	
Bloodstream	34 (39.1%)	49 (56.3%)	
Others	4 (4.6%)	8 (9.2%)	
Types of bacteria, n (%)			< 0.001*
Klebsiella pneumoniae	22 (25.3%)	26 (29.9%)	
Pseudomonas aeruginosa	20 (23.0%)	14 (16.1%)	
Acinetobacter baumannii	18 (20.7%)	6 (6.9%)	
Escherichia coli	12 (13.8%)	31 (35.6%)	
Stenotrophomonas maltophilia	12 (13.8%)	0 (0%)	
Others	3 (3.4%)	10 (11.5%)	
Multidrug resistant organisms, n (%)	71 (81.6%)	67 (77%)	0.574*
Antibiotic combination therapy, n (%)	36 (41.4%)	38 (43.7%)	0.878*
Drug resistance rates			
Quinolones	44 (50.6%)	5 (5.7%)	< 0.001*
Aminoglycosides	35 (40.2%)	50 (57.5%)	0.034*
Piperacillin-tazobactam	64 (73.6%)	16 (18.4%)	< 0.001*
Tigecycline	42 (48.3%)	26 (29.9%)	0.02*
Cephalosporin	52 (59.8%)	14 (16.1%)	< 0.001*
Sulfonamides	51 (58.6%)	63 (72.4%)	0.079

*P values are statistically significant.

### Analysis of risk factors associated with acquisition of CRGNB infection

**Table 3** showed that in univariate analysis, engraftment time of platelet > 20 days (*P*=0.002), engraftment time of neutrophil > 14 days (*P*=0.030), inappropriate empiric antimicrobial therapy (*P*=0.002), use of carbapenems > 3 days 1 month prior to infection (*P*=0.000), mechanical ventilation (*P*=0.004), admission to ICU after transplantation (*P*=0.000), and platelet transfusion volume >10 PE (*P*=0.001) were associated with the acquisition of CRGNB. In Logistic multivariate analysis, only inappropriate empiric antimicrobial therapy (HR=2.679, 95% CI 1.302-5.511, *P*=0.007), use of carbapenems > 3 days 1 month prior to infection (HR=2.988, 95% CI 1.398-6.388, *P*=0.005), admission to ICU after transplantation (HR=2.489, 95% CI 1.082-5.724, *P*=0.032) and platelet transfusion volume >10 PE (HR=2.697, 95% CI 1.274-5.711, *P*=0.010) were identified as independent risk factors for CRGNB acquisition.

**Table 3:** Univariate and multivariate analysis of CRGNB acquisition.

Variable	Univariate analysis		Multivariate analysis	
	OR (95% CI)	*P*	OR (95% CI)	*P*
Sex	0.827 (0.452,1.513)	0.538		
Age	1.009 (0.986,1.033)	0.433		
Primary disease	0.922 (0.751,1.131)	0.435		
Infection sites	1.399 (0.859,2.281)	0.178		
Types of bacteria	1.13 (0.946,1.351)	0.178		
Interval from diagnosis to transplantation > 180 days	1.153 (0.578,2.297)	0.686		
History of relapse/refractory state	1.433 (0.678,3.026)	0.346		
Time of platelet engraftment > 20 days	2.758 (1.453,5.234)	0.002*	1.582 (0.719,3.485)	0.255
Time of neutrophil engraftment > 14 days	3.118 (1.677,5.794)	0.030*	1.35 (0.646,2.824)	0.425
Acute GVHD	1.096 (0.605,1.987)	0.762		
Urethral catheterization	2.261 (0.954,5.36)	0.064		
Hospital-acquired infection	0.353 (0.09,1.377)	0.134		
Inappropriate empiric antimicrobial treatment	2.703 (1.46,5.004)	0.002*	2.679 (1.302,5.511)	0.007*
Use of carbapenems > 3 days 1 month prior to infection	3.843 (1.954,7.56)	0.000*	2.988 (1.398,6.388)	0.005*
Renal replacement therapy	3.071 (0.313,30.12)	0.335		
Mechanical ventilation	3.868 (1.555,9.622)	0.004*	1.92 (0.62,5.948)	0.258
Special immunosuppressant^#^	1.917 (0.967,3.798)	0.062		
Admission to ICU after transplantation	4.625 (2.24,9.551)	0.000*	2.489 (1.082,5.724)	0.032*
Hospital stay > 30 days	0.924 (0.425,2.011)	0.843		
Platelet transfusion volume > 10 PE	3.190 (1.648,6.173)	0.001*	2.697 (1.274,5.711)	0.010*

GVHD, graft-versus-host disease; ICU, intensive care unit; OR, odds ratio; CI, confidence interval.

^#^Special immunosuppressants here mainly refer to ruxolitinib and balliximab.

*P values are statistically significant.

### Analysis of risk factors associated with death from CRGNB infection

The comparison between death group and survival group was shown in **Table 4**. Gender (*P*=0.006), infection sites (*P*=0.001), interval from diagnosis to transplantation > 180 days (*P*=0.001), renal replacement therapy (*P*=0.018), mechanical ventilation (*P*=0.001), septic shock (*P*=0.000), platelet count < 20 × 10^9^/L (*P*=0.004), albumin < 30 g/L (*P*=0.008), total bilirubin > 34.2 μmol/L (*P*=0.007) and procalcitonin > 5 μg/L (*P*=0.001) at the beginning of infection were significantly different between the two groups in univariate analysis. In Cox multivariate analysis, interval from diagnosis to transplantation >180 days (HR=7.886, 95% CI 2.624-23.703, *P*=0.000), septic shock (HR=6.182, 95% CI 2.605-14.671, *P*=0.000), platelet count < 20 × 10^9^/L (HR=2.615, 95% CI 1.152-5.934, *P*=0.022) and total bilirubin > 34.2 μmol/L (HR=7.348, 95% CI 2.966-18.202, *P*=0.000) at the initial stage of infection were 4 independent risk factors associated with mortality.

**Table 4:** Univariate and multivariate analysis of risk factors for 100-day mortality of CRGNB infection.

Variable	Univariate analysis		Multivariate analysis	
	HR (95% CI)	*P*	HR (95% CI)	*P*
Age > 50 years	0.546 (0.214,1.394)	0.206		
Male	2.752 (1.344,5.634)	0.006*	1.303 (0.515,3.298)	0.576
Infection sites	0.371 (0.207,0.666)	0.001*	1.216(0.557,2.657)	0.624
Interval from diagnosis to transplantation > 180 days	3.692 (1.702,8.008)	0.001*	7.886 (2.624,23.703)	0.000*
Acute GVHD	0.864 (0.468,1.596)	0.641		
Inappropriate empiric antimicrobial treatment	1.166 (0.611,2.227)	0.641		
Use of carbapenems > 3 days 1 month prior to infection	1.353 (0.599,3.054)	0.467		
Renal replacement therapy	4.325 (1.284,14.572)	0.018*	1.387 (0.296,6.508)	0.678
Mechanical ventilation	3.042 (1.624,5.701)	0.001*	2.162 (0.923,5.066)	0.076
Septic shock	5.027 (2.69,9.395)	0.000*	6.182 (2.605,14.671)	0.000*
Indicators within 24 hours of infection				
Neutrophil count < 0.5×10^9^/L	1.060 (0.573,1.959)	0.853		
Platelet count < 20×10^9^/L	2.479 (1.34,4.586)	0.004*	2.615 (1.152,5.934)	0.022*
Procalcitonin > 5 μg/L	2.761 (1.482,5.143)	0.001*	1.995 (0.928,4.288)	0.077
Albumin < 30 g/L	2.32 (1.249,4.311)	0.008*	1.065 (0.485,2.337)	0.875
Total bilirubin > 34.2 μmol/L	2.489 (1.283,4.83)	0.007*	7.348 (2.966,18.202)	0.000*
Creatinine > 177 μmol/L	2.790 (0.383,20.30)	0.311		
Antibiotic combination therapy	1.234 (0.667,2.28)	0.503		
Engraftment period > 20 days	1.593 (0.825,3.078)	0.166		

GVHD, graft-versus-host disease; HR, hazard ratio; CI, confidence interval.

*P values are statistically significant.

**Figure 2:**
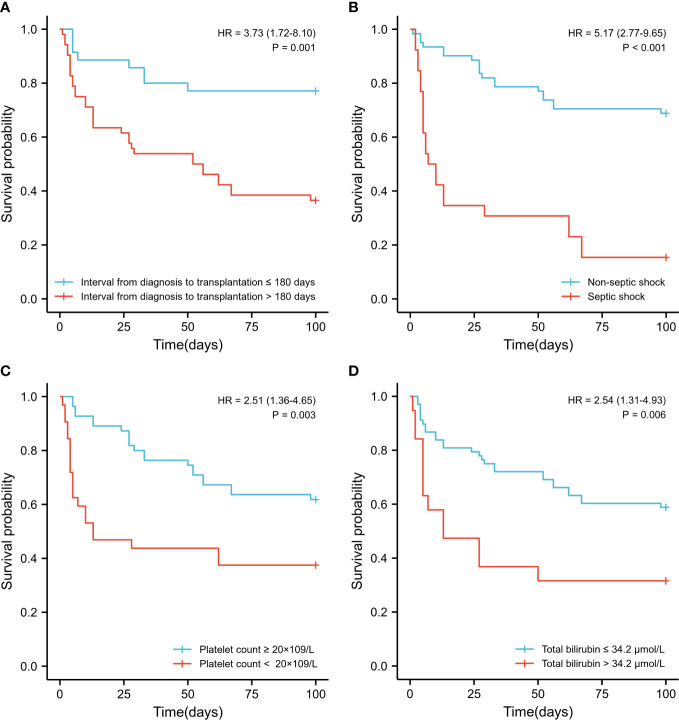
Survival comparison of patients with independent risk factors (Kaplan-Meier curve). **(A)** Time interval from diagnosis to transplantation >180 days vs. < 180 days (36.5% vs. 77.1%, *P*=0.001); **(B)** Septic shock vs. non- septic shock (15.4% vs. 68.9%, *P*<0.001); **(C)** Platelet count < 20 × 10^9^/L vs. > 20 × 10^9^/L (37.5% vs. 61.8%, *P*=0.003) at the initial stage of infection; **(D)** Total bilirubin > 34.2 μmol/L vs. < 34.2 μmol/L (31.6% vs. 58.8%, *P*=0.006) at the initial stage of infection.

The Kaplan-Meier curves of each independent risk factor were shown in **Figure 2**. Patients with interval from diagnosis to transplantation > 180 days (36.5% vs. 77.1%, *P*=0.001), septic shock (15.4% vs. 68.9%, *P*<0.001), platelet count < 20 × 10^9^/L (37.5% vs. 61.8%, *P*=0.003) and total bilirubin > 34.2 μmol/L (31.6% vs. 58.8%, *P*=0.006) at the initial stage of infection had significantly lower survival rates.

**Figure 3:**
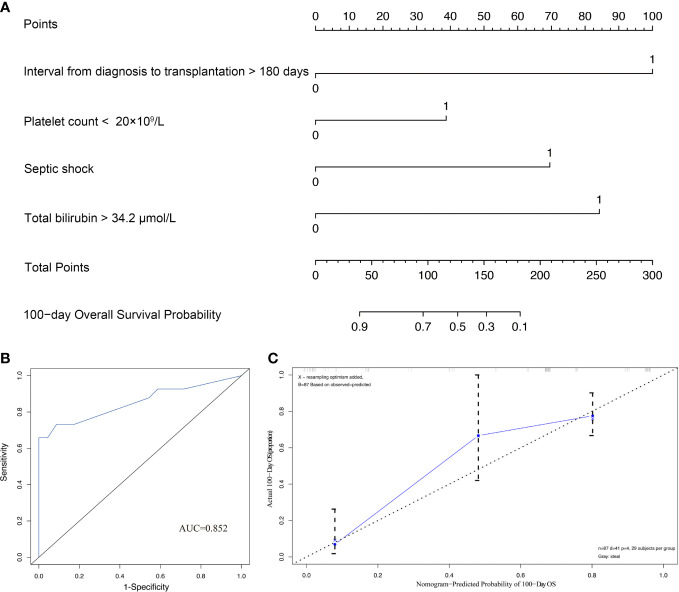
Construction and validation of prediction model for overall survival (OS). **(A)** Establishment of OS nomogram; **(B)** Receiver operating characteristics curve (ROC) of OS nomogram model; **(C)** Calibration plots of OS nomogram model.

In order to quantitatively predict the 100-day survival rate of HSCT recipients with CRGNB infection, the above independent factors were used to establish a nomogram (**Figure 3A**), and the C-index was 0.806. Meanwhile, the calibration plot also confirmed a good consistency between the predicted value of the normograph and the actual OS of CRGNB patients (**Figures 3B, C**).

## Discussion

Although global attention is now focused on COVID-19, carbapenem resistance remains a serious threat to international public health, and can even be considered an “underestimated silent pandemic”. Infections caused by GNB, particularly CRGNB, are becoming increasingly problematic in HSCT recipients (Satlin et al., 2014; Su et al., 2021).

In this work, we revealed the crude infection rate of CRGNB in HSCT recipients was 7.4% (87/1173). It is well-known that geographical differences in GNB resistance are striking. In some researches, including HSCT patients, the incidence of CRGNB ranged from 0% in Sweden (Blennow et al., 2014) to 4.7-5.8% in the United States (Satlin et al., 2016; See et al., 2016) and 20.9% in Italy (Trecarichi et al., 2015). In general, carbapenem resistance in Asia was more serious than that in Europe and the United States. Further analysis showed that lung was the most common site of infection, followed by bloodstream. Here, the incidence of CRGNB bacteremia was 2.90% (34/1173), lower than the 8.10% reported by Ding LM et al. (Ding et al., 2021). This may be related to patients’ refusal to repeatedly draw blood due to personal factors such as illness, financial or psychological pressure, which leads to limited blood culture times and low positive rates. Unlike the invasive operation of blood collection, the method of obtaining sputum culture samples was relatively easy to be accepted, so the detection rate of pulmonary infection in our data was higher. The mortality of CRGNB infection calculated here was 47.1%, much higher than that of non-CRGNB infection group (25.3%), and this difference was similar to previous reports (Averbuch et al., 2017; Vinker-Shuster et al., 2019).

As for antibiotic sensitivity, our data indicated that the resistance rates of CRGNB infected by HSCT recipients to commonly used quinolones, cephalosporins, sulfonamides and piperacillin-tazobactam were all over 50%, only relatively sensitive to some aminoglycosides and TGC, but the situation was not optimistic either. Studies have suggested that TGC has good *in vitro* activity against CRGNB strains, with a sensitivity to carbapenem resistant Klebsiella pneumoniae and Escherichia coli of 94.2% and 100%, respectively. Moreover, it is also effective for most ventilator-associated pneumonia caused by CRAB. However, a meta-analysis, which included 13 randomized clinical trials, showed that TGC was positively associated with increased mortality (Chen et al., 2020). In particular, TGC is a bacteriostatic agent that cannot directly kill bacteria, which severely limits its therapeutic reactivity in immunosuppressed hosts (Satlin et al., 2014). Fortunately, the introduction of some new antibiotics in recent years has greatly improved the therapeutic effect of CRGNB infection. For example, Ceftolozane/tazobactam (C/T) was proved to have good activity against CRPA. In addition, compared with polymyxins and aminoglycosides, it can bring higher clinical cure rate and less acute kidney injury (Pogue et al., 2020). But even so, the activity of C/T against Acinetobacter and Stenotrophomonas maltophilia is still very limited (Yahav et al., 2020). Ceftazidime avibactam (CTV) is a β-lactam/β-lactamase inhibitor combination. At present, many investigations have confirmed that CTV has excellent performance on CRGNB, especially CRKP, infected in the population after HSCT and solid organ transplantation (Chen et al., 2021; Clerici et al., 2021). However, in an *in vitro* experiment, researchers found that CRGNB isolated from patients’ blood and stool samples was resistant to CTV at a rate of 91% (Niyazi et al., 2022). Furthermore, CTV has not yet shown efficacy on CRPA and CRAB (Chalhoub et al., 2018; Sanz-García et al., 2018).

Meropenem-vaborbactam (MEV) is a combination of meropenem and vaborbactam, a new generation β-lactamase inhibitor. Alosaimi S et al. pointed out in a multicenter study from the real world that MEV has positive clinical results and safety in GNB infections, including CRE and CRPA (Alosaimy et al., 2021).

Imipenem/cilastatin/relebactam (Recarbrio™) is an intravenously administered combination of the carbapenem imipenem, the renal dehydropeptidase-I inhibitor cilastatin, and the novel β-lactamase inhibitor relebactam. It has been approved for the anti-infective treatment of CRE and CRPA (Heo, 2021). However, the addition of relebactam does not enhance the sensitivity of imipenem to Acinetobacter baumannii and Stenotrophomonas maltophilia (Principe et al., 2022). Cefiderocol is a novel siderophore cephalosporin with potent *in vitro* activity against gram-negative pathogens including MDR Enterobacteriaceae and non-fermenting organisms, such as Pseudomonas aeruginosa, Acinetobacter baumannii, Burkholderia cepacia, and Stenotrophomonas maltophilia (Abdul-Mutakabbir et al., 2020).

In addition to the above-mentioned “new antibiotics”, some relatively “ancient” preparations have also entered people’s vision again. For example, colistin, as an antibacterial agent started to be used in the 1950s, is active against a variety of GNB such as Acinetobacter baumannii, Pseudomonas aeruginosa, Escherichia coli and Klebsiella pneumoniae. Recently, because of the emergence of “superbacteria” that are resistant to other antibiotics, colistin has been revived as a salvage treatment for MDR and XDR GNB infections (El-Sayed Ahmed et al., 2020). It should be noted that on account of the narrow therapeutic window, the nephrotoxicity and neurotoxicity caused by colistin still need special attention. Interestingly, a report from Pakistan in 2021 demonstrated that even in patients with impaired renal function, colistin can effectively eliminate bacteremia, with minimal and reversible adverse reactions (Babar et al., 2021). Fosfomycin has high activity to Enterobacteriaceae, but it is ineffective to non-fermentative bacteria due to inherent resistance (Samonis et al., 2010), and its sensitivity to anaerobic bacteria is also very limited (Boyanova, 2015). Whereas, in some studies, fosfomycin has been shown to reduce the MIC of bacteria to other antibiotics, thereby restoring their sensitivity to non-fermenting bacteria (Mikhail et al., 2019). Unfortunately, owing to the actual factors such as the time when drugs are approved for marketing, drug prices, medical insurance system, and patients’ economic affordability, many antibiotics have not been widely used in our patients. At the same time, some corresponding drug sensitivity tests were not carried out, resulting in incomplete data that could not be included in subsequent statistical analysis.

Not surprisingly, the mortality of HSCT recipients in the CRGNB infected group was significantly higher than that in the matched non-CRGNB group (47.1% vs. 25.3%). Further multivariate analysis revealed that the interval from diagnosis to transplantation > 180 days, septic shock, low platelet count and high bilirubin level at the initial stage of infection were independent risk factors for death. In previous reports, we have described the adverse effects of extended transplant interval: persistent disease state, multiple chemotherapy treatments, low immunity, poor body function, delayed donor cells implantation, etc (Liu et al., 2022). This is why both domestic and international guidelines unanimously recommend early transplantation for eligible patients (Blackburn et al., 2019). However, the fact is, due to age, personal financial situation, family support, medical environment, donor factors, etc., there are still many patients cannot receive HSCT within the optimal time range, which affects the prognosis to some extent.

The conclusion that septic shock was an independent risk factor associated with mortality in CRGNB patients is similar to previous reports (Qiao et al., 2017; Aguado et al., 2018). This seems to be expected, since septic shock is one of the most critical indicators of disease severity. This finding emphasizes the importance of early identification and treatment, which may improve survival for patients with septic shock and the resulting respiratory and heart failure (Wu et al., 2021).

The primary risk of extremely low platelet count at the initial stage of infection is bleeding, especially in important organs and intracranial hemorrhage. Meanwhile, it is also a side warning of patients’ insufficient hematopoietic recovery ability. Infection, severe bleeding, and delayed hematopoietic reconstruction undoubtedly bring great obstacles to the rehabilitation of patients after transplantation.

A high level of serum total bilirubin means poor liver function, which may be caused by underlying liver-related diseases (such as viral hepatitis), or drug-induced liver damage. But either way, it will lay a “time bomb” for the future development of hepatic veno-occlusive disease (HVOD) or liver rejection (Yoon et al., 2021). HVOD is one of the most serious complications after HSCT, and the mortality of severe patients can be up to 50% (Bajwa et al., 2017). The main clinical manifestations include fluid retention, ascites, weight gain, jaundice, hepatomegaly and pain, and even multiple organ failure. Researches learned that the risk factors of HVOD involve multiple aspects, such as patient’s primary disease, age, complications, increased serum ferritin, acute renal failure, transplantation type, pretreatment regimen, and anti-GVHD drugs (Corbacioglu et al., 2019). Therefore, avoiding hepatotoxic drugs as much as possible and strengthening liver function protection to reduce the occurrence of HVOD have a positive effect on improving the survival rate of patients after transplantation. Currently, ursodeoxycholic acid (UDCA) and defibrotide (DF) have been recommended for the prevention of HVOD (Carreras, 2012; Dignan et al., 2013). DF is also approved for the treatment of HVOD by the United States Food and Drug Administration (FDA) and the European Union (Corbacioglu et al., 2019). Although some other drugs such as prostaglandin E1 (PGE1) have also been reported to have therapeutic effects on HVOD, their exact efficacy still needs to be further verified by prospective clinical studies (Dignan et al., 2013).

Admittedly, there are some limitations to our study. First, due to the retrospective nature, we cannot perform molecular analyses to identify the types of carbapenemases produced by CRGNB organisms. Second, this is a single-center cohort study, so the results may not be popularized to other regions. Third, as previously mentioned, clinical data and efficacy assessments for some novel antibiotics are not yet available. However, this exactly reflects the actual problems faced by different regions of the world in the utilization of medical resources at this stage. By reviewing a large number of literatures, we summarized the relevant information on the use of antibiotics against CRGNB, hoping to provide reference for medical staff in the future treatment options.

## Conclusion

In short, HSCT recipients are prone to CRGNB infection, and the drug resistance rate and mortality rate are extremely high. Interval from diagnosis to transplantation >180 days, septic shock, platelet count < 20 × 10^9^/L and total bilirubin > 34.2 μmol/L at the initial stage of infection were 4 independent risk factors associated with death. Shortening the transitional waiting time of transplant population, strengthening liver function screening and protection, appropriate platelet transfusion to reduce bleeding risk, and early identification and timely correction of shock will help improve the prognosis of patients.

## Data availability statement

The original contributions presented in the study are included in the article/supplementary material. Further inquiries can be directed to the corresponding author.

## Author contributions

YJ and YFL were involved in the study conception and design. YunL, YiL, ZY and XC participated in the data management. All authors participated in the interpretation and statistical analysis of data. YJ wrote the draft and YFL revised it. All authors contributed to the article and approved the submitted version.

## References

[B1] Abdul-Mutakabbir J. C. Alosaimy S. Morrisette T. Kebriaei R. Rybak M. J. (2020). Cefiderocol: a novel siderophore cephalosporin against multidrug-resistant gram-negative pathogens. Pharmacotherapy. 40, 1228–1247. doi: 10.1002/phar.2476 33068441

[B2] Aguado J. M. Silva J. T. Fernandez-Ruiz M. Cordero E. Fortun J. Gudiol C.. (2018). Management of multidrug resistant gram-negative bacilli infections in solid organ transplant recipients: SET/GESITRA-SEIMC/REIPI recommendations. Transplant. Rev. (Orlando). 32, 36–57. doi: 10.1016/j.trre.2017.07.001 28811074

[B3] Alosaimy S. Lagnf A. M. Morrisette T. Scipione M. R. Zhao J. J. Jorgensen S. C. J.. (2021). Real-world, multicenter experience with meropenem-vaborbactam for gram-negative bacterial infections including carbapenem-resistant enterobacterales and pseudomonas aeruginosa. Open Forum Infect. Dis. 8, ofab371. doi: 10.1093/ofid/ofab371 34430671 PMC8378588

[B4] Averbuch D. Tridello G. Hoek J. Mikulska M. Akan H. Yanez San Segundo L.. (2017). Antimicrobial resistance in gram-negative rods causing bacteremia in hematopoietic stem cell transplant recipients: Intercontinental prospective study of the infectious diseases working party of the European bone marrow transplantation group. Clin. Infect. Dis. 65, 1819–1828. doi: 10.1093/cid/cix646 29020364

[B5] Babar Z. U. Dodani S. K. Nasim A. (2021). Treatment outcome and adverse effects of colistin in adult patients with carbapenem-resistant gram-negative bacteremia from Pakistan. Int. J. Infect. Dis. 106, 171–175. doi: 10.1016/j.ijid.2021.03.004 33705852

[B6] Babiker A. Clarke L. G. Saul M. Gealey J. A. Clancy C. J. Nguyen M. H.. (2021). Changing epidemiology and decreased mortality associated with carbapenem-resistant gram-negative bacteri-2017. Clin. Infect. Dis. 73, e4521–e4530. doi: 10.1093/cid/ciaa1464 32990319 PMC8662792

[B7] Bajwa R. P. S. Mahadeo K. M. Taragin B. H. Dvorak C. C. McArthur J. Jeyapalan A.. (2017). Consensus report by pediatric acute lung injury and sepsis investigators and pediatric blood and marrow transplantation consortium joint working committees: supportive care guidelines for management of veno-occlusive disease in children and adolescents, part 1: Focus on investigations, prophylaxis, and specific treatment. Biol. Blood Marrow Transplant. 23, 1817–1825. doi: 10.1016/j.bbmt.2017.07.021 28754544

[B8] Blackburn L. M. Bender S. Brown S. (2019). Acute leukemia: Diagnosis and treatment. Semin. Oncol. Nurs. 35, 150950. doi: 10.1016/j.soncn.2019.150950 31757585

[B9] Blennow O. Ljungman P. Sparrelid E. Mattsson J. Remberger M. (2014). Incidence, risk factors, and outcome of bloodstream infections during the pre-engraftment phase in 521 allogeneic hematopoietic stem cell transplantations. Transpl Infect. Dis. 16, 106–114. doi: 10.1111/tid.12175 24372809

[B10] Boyanova L. (2015). Susceptibility of anaerobes to fusidic acid and fosfomycin. Int. J. Antimicrob. Agents. 45, 560–561. doi: 10.1016/j.ijantimicag.2015.02.003 25769788

[B11] Carreras E. (2012). Early complications after HSCT. ebmt-esh handbook on haematopoietic stem cell transplantation. 176e195. Available at: http://ebmtonline.forumservice.net/media/11/tex/content_alt/EBMT_Handbook2012_CHAP11.pdf.

[B12] Chalhoub H. Saenz Y. Nichols W. W. Tulkens P. M. Van Bambeke F. (2018). Loss of activity of ceftazidime-avibactam due to MexAB-OprM efflux and overproduction of AmpC cephalosporinase in pseudomonas aeruginosa isolated from patients suffering from cystic fibrosis. Int. J. Antimicrob. Agents. 52, 697–701. doi: 10.1016/j.ijantimicag.2018.07.027 30081137

[B13] Chen F. Shen C. Pang X. Zhang Z. Deng Y. Han L.. (2020). Effectiveness of tigecycline in the treatment of infections caused by carbapenem-resistant gram-negative bacteria in pediatric liver transplant recipients: A retrospective study. Transpl Infect. Dis. 22, e13199. doi: 10.1111/tid.13199 31627248

[B14] Chen F. Zhong H. Yang T. Shen C. Deng Y. Han L.. (2021). Ceftazidime-avibactam as salvage treatment for infections due to carbapenem-resistant klebsiella pneumoniae in liver transplantation recipients. Infect. Drug Resist. 14, 5603–5612. doi: 10.2147/IDR.S342163 34992387 PMC8710070

[B15] Chinese Society of Hematology Chinese Medical Association (2019). Chinese Guidelines for diagnosis and treatment of myelodysplastic syndrome). Zhonghua Xue Ye Xue Za Zhi. 40, 89–97. doi: 10.3760/cma.j.issn.0253-2727.2019.02.001 30831622 PMC7342655

[B16] Chinese Society of Hematology Chinese Medical Association Chinese Medical Doctor Association (2020). Chinese Guidelines for the clinical application of antibacterial drugs for agranulocytosis with feve). Zhonghua Xue Ye Xue Za Zhi. 41, 969–978. doi: 10.3760/cma.j.issn.0253-2727.2020.12.001 33445842 PMC7840550

[B17] Clerici D. Oltolini C. Greco R. Ripa M. Giglio F. Mastaglio S.. (2021). The place of ceftazidime/avibactam and ceftolozane/tazobactam for therapy of haematological patients with febrile neutropenia. Int. J. Antimicrob. Agents. 57, 106335. doi: 10.1016/j.ijantimicag.2021.106335 33838223

[B18] Corbacioglu S. Jabbour E. J. Mohty M. (2019). Risk factors for development of and progression of hepatic veno-occlusive disease/sinusoidal obstruction syndrome. Biol. Blood Marrow Transplant. 25, 1271–1280. doi: 10.1016/j.bbmt.2019.02.018 30797942

[B19] Dignan F. L. Wynn R. F. Hadzic N. Karani J. Quaglia A. Pagliuca A.. (2013). BCSH/BSBMT guideline: Diagnosis and management of veno-occlusive disease (sinusoidal obstruction syndrome) following haematopoietic stem cell transplantation. Br. J. Haematol. 163, 444–457. doi: 10.1111/bjh.12558 24102514

[B20] Ding L. M. Song X. L. Wang X. G. Peng Y. Chen Y. R. Jin L.. (2021). Analysing pathogenic bacterial spectrum and drug resistance of bloodstream infection in patients with allogeneic hematopoietic stem cell transplantation. Zhonghua Xue Ye Xue Za Zhi. 42, 807–813. doi: 10.3760/cma.j.issn.0253-2727.2021.10.003 34788919 PMC8607017

[B21] Doi Y. (2019). Treatment options for carbapenem-resistant gram-negative bacterial infections. Clin. Infect. Dis. 69, S565–S575. doi: 10.1093/cid/ciz830 31724043 PMC6853760

[B22] El-Sayed Ahmed M. A. E. Zhong L. L. Shen C. Yang Y. Doi Y. Tian G. B. (2020). Colistin and its role in the era of antibiotic resistance: An extended revie-2019). Emerg. Microbes Infect. 9, 868–885. doi: 10.1080/22221751.2020.1754133 32284036 PMC7241451

[B23] Hematology Oncology Committee Chinese Anti-Cancer Association Leukemia & Lymphoma Group Chinese Society of Hematology Chinese Medical Association (2021). Chinese Guidelines for diagnosis and treatment of adult acute lymphoblastic leukemi). Zhonghua Xue Ye Xue Za Zhi. 42, 705–716. doi: 10.3760/cma.j.issn.0253-2727.2021.09.001 34753224 PMC8607046

[B24] Heo Y. A. (2021). Imipenem/Cilastatin/Relebactam: A review in gram-negative bacterial infections. Drugs. 81, 377–388. doi: 10.1007/s40265-021-01471-8 33630278 PMC7905759

[B25] Hoppe R. T. Advani R. H. Ai W. Z. Ambinder R. F. Armand P. Bello C. M.. (2020). Hodgkin Lymphoma, version 2.2020, NCCN clinical practice guidelines in oncology. J. Natl. Compr. Canc Netw. 18, 755–781. doi: 10.6004/jnccn.2020.0026 32502987

[B26] Horan T. C. Andrus M. Dudeck M. A. (2018). CDC/NHSN surveillance definition of health care-associated infection and criteria for specific types of infections in the acute care setting. Am. J. Infect. Control. 36, 309–332. doi: 10.1016/j.ajic.2008.03.002 18538699

[B27] Huo Y. Y. Pang A. M. Cheng T. (2020). Advance in hematopoietic and immune reconstitution of allogeneic stem cell transplantation. Zhonghua Xue Ye Xue Za Zhi. 41, 958–963. doi: 10.3760/cma.j.issn.0253-2727.2020.11.018 33333706 PMC7767801

[B28] Khaddour K. Hana C. K. Mewawalla P. (2022). Hematopoietic stem cell transplantation (Treasure Island (FL: StatPearls Publishing). 30725636

[B29] Leukemia & Lymphoma Group Chinese Society of Hematology Chinese Medical Association (2021). Chinese Guidelines for the diagnosis and treatment of adult acute myeloid leukemia (not APL) (2021). Zhonghua Xue Ye Xue Za Zhi. 42, 617–623. doi: 10.3760/cma.j.issn.0253-2727.2021.08.001 34547865 PMC8501285

[B30] Liu Y. F. Liu Y. Chen X. Jia Y. (2022). Epidemiology, drug resistance, and risk factors for mortality among hematopoietic stem cell transplantation recipients with hospital-acquired klebsiella pneumoniae infections: A single-center retrospective study from China. Infect. Drug Resist. 15, 5011–5021. doi: 10.2147/IDR.S376763 36065276 PMC9440706

[B31] Mikhail S. Singh N. B. Kebriaei R. Rice S. A. Stamper K. C. Castanheira M.. (2019). Evaluation of the synergy of ceftazidime-avibactam in combination with meropenem, amikacin, aztreonam, colistin, or fosfomycin against well-characterized multidrug-resistant klebsiella pneumoniae and pseudomonas aeruginosa. Antimicrob. Agents Chemother. 63, e00779–19. doi: 10.1128/AAC.00779-19 31182535 PMC6658738

[B32] Mikulska M. Del Bono V. Bruzzi P. Raiola A. M. Gualandi F. Van Lint M. T.. (2012). Mortality after bloodstream infections in allogeneic haematopoietic stem cell transplant (HSCT) recipients. Infection. 40, 271–278. doi: 10.1007/s15010-011-0229-y 22187340

[B33] Mikulska M. Viscoli C. Orasch C. Livermore D. M. Averbuch D. Cordonnier C.. (2014). Aetiology and resistance in bacteraemias among adult and paediatric haematology and cancer patients. J. Infect. 68, 321–331. doi: 10.1016/j.jinf.2013.12.006 24370562

[B34] Niyazi D. Micheva I. Savova D. Stoeva T. (2022). *In vitro* activity of ceftazidime-avibactam against ESBL producing and carbapenem-resistant gram–negative bacteria recovered from blood and fecal samples of patients after hematopoietic stem-cell transplantation. Sciforum. doi: 10.3390/eca2022-12691

[B35] Omrani A. S. Almaghrabi R. S. (2017). Complications of hematopoietic stem cell transplantation: Bacterial infections. Hematol. Oncol. Stem Cell Ther. 10, 228–232. doi: 10.1016/j.hemonc.2017.05.018 28692817

[B36] Ortega M. Rovira M. Almela M. Marco F. de la Bellacasa J. P. Martinez J. A.. (2005). Bacterial and fungal bloodstream isolates from 796 hematopoietic stem cell transplant recipients between 1991 and 2000. Ann. Hematol. 84, 40–46. doi: 10.1007/s00277-004-0909-0 15480665

[B37] Pogue J. M. Kaye K. S. Veve M. P. Patel T. S. Gerlach A. T. Davis S. L.. (2020). Ceftolozane/Tazobactam vs polymyxin or aminoglycoside-based regimens for the treatment of drug-resistant pseudomonas aeruginosa. Clin. Infect. Dis. 71, 304–310. doi: 10.1093/cid/ciz816 31545346

[B38] Poutsiaka D. D. Price L. L. Ucuzian A. Chan G. W. Miller K. B. Snydman D. R. (2007). Blood stream infection after hematopoietic stem cell transplantation is associated with increased mortality. Bone Marrow Transplant. 40, 63–70. doi: 10.1038/sj.bmt.1705690 17468772

[B39] Principe L. Lupia T. Andriani L. Campanile F. Carcione D. Corcione S.. (2022). Microbiological, clinical, and PK/PD features of the new anti-gram-negative antibiotics: Beta-lactam/beta-lactamase inhibitors in combination and cefiderocol-an all-inclusive guide for clinicians. Pharm. (Basel). 15, 463. doi: 10.3390/ph15040463 PMC9028825 35455461

[B40] Qiao B. Wu J. Wan Q. Zhang S. Ye Q. (2017). Factors influencing mortality in abdominal solid organ transplant recipients with multidrug-resistant gram-negative bacteremia. BMC Infect. Dis. 17, 171. doi: 10.1186/s12879-017-2276-1 28241746 PMC5327527

[B41] Red Blood Cell Disease (Anemia) Group Chinese Society of Hematology Chinese Medical Association (2017). Chinese Expert consensus on the diagnosis and treatment of aplastic anemi). Zhonghua Xue Ye Xue Za Zhi. 38, 1–5. doi: 10.3760/cma.j.issn.0253-2727.2017.01.001 28219216 PMC7348398

[B42] Sahitya D. S. K. Jandiyal A. Jain A. Senapati J. Nanda S. Aggarwal M.. (2021). Prevention and management of carbapenem-resistant enterobacteriaceae in haematopoietic cell transplantation. Ther. Adv. Infect. Dis. 8, 20499361211053480. doi: 10.1177/20499361211053480 34733507 PMC8558808

[B43] Samonis G. Maraki S. Rafailidis P. I. Kapaskelis A. Kastoris A. C. Falagas M. E. (2010). Antimicrobial susceptibility of gram-negative nonurinary bacteria to fosfomycin and other antimicrobials. Future Microbiol. 5, 961–970. doi: 10.2217/fmb.10.47 20521939

[B44] Sanz-García F. Hernando-Amado S. Martínez J. L. (2018). Mutation-driven evolution of pseudomonas aeruginosa in the presence of either ceftazidime or ceftazidime/avibactam. Antimicrob. Agents Chemother. 62, e01379–e01318. doi: 10.1128/AAC.01379-18 30082283 PMC6153820

[B45] Satlin M. J. Cohen N. Ma K. C. Gedrimaite Z. Soave R. Askin G.. (2016). Bacteremia due to carbapenem-resistant enterobacteriaceae in neutropenic patients with hematologic malignancies. J. Infect. 73, 336–345. doi: 10.1016/j.jinf.2016.07.002 27404978 PMC5026910

[B46] Satlin M. J. Jenkins S. G. Walsh T. J. (2014). The global challenge of carbapenem-resistant enterobacteriaceae in transplant recipients and patients with hematologic malignancies. Clin. Infect. Dis. 58, 1274–1283. doi: 10.1093/cid/ciu052 24463280 PMC4038783

[B47] See I. Freifeld A. G. Magill S. S. (2016). Causative organisms and associated antimicrobial resistance in healthcare-associated, central line-associated bloodstream infections from oncology setting-2012. Clin. Infect. Dis. 62, 1203–1209. doi: 10.1093/cid/ciw113 26936664 PMC4894695

[B48] Shields R. K. Potoski B. A. Haidar G. Hao B. Doi Y. Chen L.. (2016). Clinical outcomes, drug toxicity, and emergence of ceftazidime-avibactam resistance among patients treated for carbapenem-resistant enterobacteriaceae infections. Clin. Infect. Dis. 63, 1615–1618. doi: 10.1093/cid/ciw636 27624958 PMC5146720

[B49] Stem Cell Application Group Chinese Society of Hematology Chinese Medical Association (2020). Chinese Consensus of allogeneic hematopoietic stem cell transplantation for hematological disease (III) -acute graft-versus-host diseas). Zhonghua Xue Ye Xue Za Zhi. 41, 529–536. doi: 10.3760/cma.j.issn.0253-2727.2020.07.001 32549120 PMC7449769

[B50] Su F. Luo Y. Yu J. Shi J. Zhao Y. Yan M.. (2021). Tandem fecal microbiota transplantation cycles in an allogeneic hematopoietic stem cell transplant recipient targeting carbapenem-resistant enterobacteriaceae colonization: A case report and literature review. Eur. J. Med. Res. 26, 37. doi: 10.1186/s40001-021-00508-8 33910622 PMC8080403

[B51] Trecarichi E. M. Pagano L. Candoni A. Pastore D. Cattaneo C. Fanci R.. (2015). Current epidemiology and antimicrobial resistance data for bacterial bloodstream infections in patients with hematologic malignancies: An Italian multicentre prospective survey. Clin. Microbiol. Infect. 21, 337–343. doi: 10.1016/j.cmi.2014.11.022 25595706

[B52] Vinker-Shuster M. Stepensky P. Temper V. Shayovitz V. Masarwa R. Averbuch D. (2019). Gram-negative bacteremia in children with hematologic malignancies and following hematopoietic stem cell transplantation: Epidemiology, resistance, and outcome. J. Pediatr. Hematol. Oncol. 41, e493–e498. doi: 10.1097/MPH.0000000000001556 31318820

[B53] Wan Q. Liu H. Ye S. Ye Q. (2017). Confirmed transmission of bacterial or fungal infection to kidney transplant recipients from donated after cardiac death (DCD) donors in China: A single-center analysis. Med. Sci. Monit. 23, 3770–3779. doi: 10.12659/msm.901884 28771455 PMC5553435

[B54] Wu D. Chen C. Liu T. Jia Y. Wan Q. Peng J. (2021). Epidemiology, susceptibility, and risk factors associated with mortality in carbapenem-resistant gram-negative bacterial infections among abdominal solid organ transplant recipients: A retrospective cohort study. Infect. Dis. Ther. 10, 559–573. doi: 10.1007/s40121-021-00411-z 33611687 PMC7954940

[B55] Yahav D. Giske C. G. Gramatniece A. Abodakpi H. Tam V. H. Leibovici L. (2020). New beta-lactam-beta-lactamase inhibitor combinations. Clin. Microbiol. Rev. 34, e00115-20. doi: 10.1128/CMR.00115-20 PMC7667665 33177185

[B56] Yoon J. H. Min G. J. Park S. S. Park S. Lee S. E. Cho B. S.. (2021). Incidence and risk factors of hepatic veno-occlusive disease/sinusoidal obstruction syndrome after allogeneic hematopoietic cell transplantation in adults with prophylactic ursodiol and intravenous heparin or prostaglandin E1. Bone Marrow Transplant. 56, 1603–1613. doi: 10.1038/s41409-021-01215-y 33526915

